# Adopting a Global AMR Target within the Pandemic Instrument Will Act as a Catalyst for Action

**DOI:** 10.1017/jme.2022.101

**Published:** 2022

**Authors:** Susan Rogers Van Katwyk, Lindsay Wilson, Isaac Weldon, Steven J. Hoffman, Mathieu J.P. Poirier

**Affiliations:** 1: YORK UNIVERSITY IN TORONTO, ONTARIO, CANADA.

**Keywords:** Pandemic Treaty, One Health, Antimicrobial Resistance, Goal Setting, Global Health

## Abstract

Ensuring that life-saving antimicrobials remain available as effective treatment options in the face of rapidly rising levels of antimicrobial resistance will require a massive and coordinated global effort. Setting a collective direction for progress is the first step towards aligning global efforts on AMR. This process would be greatly accelerated by adopting *a unifying global target* — a well-defined global target that unites all countries and sectors. The proposed pandemic instrument — with its focus on prevention, preparedness and response — represents an ideal opportunity to develop and adopt a unifying global target that catalyzes global action on AMR. We propose three key characteristics of a unifying global target for AMR that — if embedded within the pandemic preparedness instrument — could rally public support, funding, and political commitment commensurate with the scale of the AMR challenge.

## Introduction

Antimicrobial resistance (AMR) is a substantial threat to human and animal health, global economies, and the environment. Increasing drug resistance is already manifesting in longer hospitalizations, higher morbidity and mortality, and rising healthcare costs associated with infections that were, until recently, considered treatable.^1^ Achieving global progress on AMR and ensuring that these life-saving drugs remain available as treatment options for future generations^2^ will require a massive and coordinated effort to implement a suite of interventions aimed at conserving antimicrobial effectiveness.

Setting a collective direction for global progress is the first step towards aligning global efforts on AMR. This process would be greatly accelerated by adopting a *unifying global target* — a defined global goal that unites all countries and sectors in their collective efforts to address AMR. Without a global target to rally support, past efforts to address AMR have lacked the necessary level of ambition and coordination to secure a future with sustainable antimicrobial use for all.^3^ Left unmitigated, the impact of AMR on global health and development, especially in low- and middle-income countries (LMICs), will be immense.^4^ AMR is often perceived as a technocratic challenge, and a focus on medical and drug development solutions ignores the important infrastructural role of antibiotics in society.^5^ A global effort to articulate a unifying global target could help rally public support, funding, and political commitment commensurate with the scale of the AMR challenge. Embedding this unifying global target for AMR within the pandemic instrument has the potential to rally this support and catalyze effective action on AMR. In this article, we identify three key characteristics of an effective unifying global target for AMR based on past experiences of unifying global targets in climate and global health domains (Box 1).

Box 1 Summary of Three Recommended Characteristics of a Unifying Global Target for AMR

1. **A unifying global target should act as a barometer of global progress across all countries and sectors**
  To ensure global collective action, a unifying global target for AMR should act as a barometer for global success and should provide an overview of collective progress across human, animal, and environmental sectors to meaningfully address AMR in ways that matter to people.
2. **A unifying global target should conceptually unite many technical perspectives as a single clearly communicated concept**
  Politicians and the public are the primary audience for a unifying global target and as such, the target must prioritize clarity and accessibility. Rather than selecting one or more technical indicators, the unifying global target should conceptually unite many technical perspectives or indicators as a single easily communicated concept that is universally valued and inherently important.
3. **A unifying global target should be action-oriented to give identifiable moments of success**
  To rally support for global action on AMR, a unifying global target must include clear steps to make progress on the goal and offer the opportunity for identifiable moments of success.

## What Is a Unifying Global Target?

A unifying global target is a high-profile goal with a particular emphasis on raising attention and catalyzing action on key global issues. Unlike the indicators, monitoring strategies, and benchmarks that emerge from the goal and allow technical experts to track progress toward it, the intended audience of a unifying global target are politicians and the general public. High-profile examples of unifying global targets are the Millennium Development Goals (MDGs), Sustainable Development Goals (SDGs), and UNAIDS’ 90-90-90 goal.^6^ Perhaps the most prominent example of a unifying global target is the objective that was legally codified within the Paris Climate Agreement, which established an overarching goal to keep global warming well below 2° Celsius compared to pre-industrial levels.^7^

Goals are powerful tools in global governance, catalyzing action on the international stage in much the same way as individual or small group action. At their core, goals act as vehicles for global norms, help to direct attention and effort towards goal-relevant activities, serve an energizing function, and motivate persistence so that efforts are extended over longer periods of time.^8^ Importantly, goals guide strategies, action plans, and other problem solving techniques,^9^ which makes them a fundamental component of successful international legal agreements and other global governance arrangements.^10^

Since goals embody and exercise various forms of power, the act of goal-setting is an inherently political process. Global goals represent a shared aspiration for the stated objective, suggesting that the goal, and the norm that it embodies, are universally desirable for the future. Translating the global goal into indicators can also pose political challenges, despite being a largely technocratic process. Indicators must be able to meaningfully measure how well a country is performing on a particular aspect of the goal.^11^ In this way, goals and their indicators project a specific understanding of reality, which may raise concerns about ‘objectivity’ and ‘universality’ when representing the world as it is and as it should be. The political process of goal setting also shapes the framing, ambition, and implementation for the goal.^12^ Without attention to the political importance of these processes, the goal could cause unintended effects by incentivizing ‘gaming’ or overemphasizing some priorities, locations, and aspect of the problem over others.^13^ Considering these political challenges, if the process of forming the goal and translating it into measurable indicators — to say nothing of the goal itself — are not inclusive and equity driven, the goal could fail to gain traction globally or fail to incentivize appropriate actions, while also exacerbating the status quo of inequalities and paternalism in global governance.^14^

### 1. A Unifying Global Target Should Act as a Barometer of Global Progress across All Countries and Sectors

In the past, some goals have been designed through the lens of international competition and have been perceived as an opportunity to rebuke or discipline of underperforming countries. Once goals are set and transformed into indicators, country actions can be measured, evaluated, ranked, and compared against the performance of others. In theory, goal setting can help countries ‘self-regulate’ or pressure laggard states toward a norm for fear of reputational consequences, while the use of progress indicators can be used to allocate resources and penalties.^15^

Given the global nature of AMR and our inability to isolate the spread of resistance to particular regions, it is essential that AMR is perceived as a global issue requiring global collective action. Attempts to adopt simple goals that can be measured at the national level in other areas of global health have failed to achieve the intended effects of the unifying global target.

This ranking approach to goal setting is out of step with the purpose of a unifying global target. Given the global nature of AMR and our inability to isolate the spread of resistance to particular regions, it is essential that AMR is perceived as a global issue requiring global collective action. Attempts to adopt simple goals that can be measured at the national level in other areas of global health have failed to achieve the intended effects of the unifying global target. The MDGs, for example, characterized as a “report card for global development”^16^ have been criticized because many countries that made substantial progress often still fell short of meeting the MDG target and were therefore perceived as having failed to meet the goal.^17^ By contrast, free riding was common among high income countries, many of whom failed to deliver on their official development assistance commitments.^18^

Although its indicators may facilitate naming and shaming, a unifying global target should primarily act as a barometer of global progress on the global issue and will ideally not even be measurable at the national level. This is the case for the Paris Agreement’s unifying target: the success or failure to maintain average global temperatures below 2°C above pre-industrial levels^19^ cannot be measured at the national level, and as such, requires all countries to remain committed and focused on the goal. Instead of ranking countries on a common indicator, the Paris Agreement draws attention to countries failing to meet their nationally defined targets, achieving the disciplining function of goal-setting, without holding countries to a set of identical targets.

Finally, for multisectoral challenges, a successful unifying global target must be applied cross sectors to show a complete picture of global successes and failures. A target that exclusively relies on data on human health may not accurately represent global progress on AMR in animal and environmental sectors. A unifying global target must aggregate the actions of diverse industries and present a single clear message. Experts may use technical indicators to assess the appropriateness of antimicrobial use in individual sectors; however, a unifying global target should portray whether action across sectors is adding up to an appropriate, significant, sufficient response. This has been done in the case of the Paris target which represents the global progress; technical indicators are used by experts to assess specific challenges around greenhouse gas concentrations, warming, emissions, ice melt, and pollution, but these do not represent the full picture of the global response. In the case of AMR, the contributions — both towards the problem and towards progress — of all One Health sectors (human, animal, and environment) need to be aggregated into a single goal.

### 2. A Unifying Global Target Should Conceptually Unite Many Technical Perspectives as a Single Clearly Communicated Concept

An effective global goal must strike a balance between accurately reflecting progress on the nuanced scientific issue of AMR while also being clear and easily understood by the primary audience: politicians and the general public. As such, a unifying global target should not be a biological indicator or other technical metric typically used by specialists for monitoring the evolution of AMR.

While global goals can include multiple indicators, the overall direction should be clear, and the specific actions that will be necessary to make them successful must be directly conveyed.^20^ In order to facilitate buy-in and increase the feasibility of a global AMR goal, the goal must be approachable and make sense for the people who are being asked to act on it. Instead of looking to technical indicators, a unifying global target should instead focus on conceptual clarity with the goal of conveying a single key message to the core audience. The 2°C goal of the Paris Agreement serves this function — it helps clarify the trajectory of progress by requiring the integration of inputs from multiple sources. To achieve this, specialists with the Intergovernmental Panel on Climate Change pull together data from technical indicators, including greenhouse gas concentrations, warming, emissions, ice melt, and pollution, among many others, into regular reports that inform the global stocktake and discussions as to whether or not the world is on track to meet the 2°C unifying global target.^21^ A similar unifying global target is needed in AMR to catalyze the synthesis of data on infections and resistance patterns across One Health sectors in order to provide the world with a clear message on the state of global progress in combatting AMR.

### 3. A Unifying Global Target Must Be Action-Oriented to Give Identifiable Moments of Success

Above all, a global AMR goal needs to act as a rallying cry around which the international community can mobilize. Currently, the dominant narrative is that AMR is a looming crisis with an extremely bleak prognosis.^22^ This is due in part to the lack of actionable goals around AMR: without a strategy to mitigate the risks associated with AMR, the problem can seem insurmountable. Research and guidance on ways to effectively communicate about AMR has emphasized the need to focus on the present need for immediate action rather than apocalyptic scenarios.^23^ Global goals that clearly identify what success looks like, and present clear actions or steps towards solving the problem can invigorate action by providing updates and offering good news stories.

Eradication goals, such as efforts to eradicate smallpox, rinderpest, and polio, have been largely successful unifying global targets, rallying substantial support over decades in part through their success stories and clearly identifiable action steps. The 2020 rallying cry to “flatten the curve” during the early COVID-19 pandemic — which referred to slowing the rate of new infections in order to reduce the peak of the epidemic curve^24^ and avoid overwhelming the healthcare system^25^ — also achieved temporary success, as it offered a clear and action oriented rationale for the restrictive measures adopted to limit the spread of COVID-19. In articulating what success looks like, the Paris Climate goal is also action oriented; however, it offers few opportunities to tell success stories due to its long end-date. A global goal for AMR should include a concrete strategy that can act as a framework for action and sustain momentum over the short-term through long-term moments of success.

## Existing Indicators for AMR Fail to Meet the Criteria for a Unifying Global Target

Various metrics for analyzing and communicating AMR priorities or thresholds have been proposed (Box 2). While each existing goal, indicator or target has merits, none that we identified would be ideal to adopt as a unifying global target. For instance, while it is useful to have an AMR indicator embedded within the SDGs, the national percentage reduction in bloodstream infections is an technical indicator that, while useful to specialists comparing countries, is not appropriate for adequately conveying the urgency of the problem to the public or for rallying multisectoral support for addressing AMR. Other proposed prescribing targets measured in Defined Daily Doses, milligrams of antimicrobials per kilogram of animal (mg/kg), milligrams of antimicrobials per Population Correction Unity (mg/PCU)^29^ are also too technical and fail to convey their inherent value or immediate relevance. While achieving these targets would create identifiable moments of success, the importance of these moments would be difficult to communicate to the general public and political leaders.

Box 2 Previously Proposed and Existing AMR Goals, Indicators and Targets

**Percent reduction in bloodstream infections** (United Nations 2020)

The Sustainable Development Goals include a target to reduce blood stream infections.

**Antibiotic footprint** (Limmathurotsakul et al. 2019)

In line with climate change ‘carbon footprint,’ the ‘antibiotic footprint’ has been proposed as a communication tool to illustrate the magnitude of antibiotic use across sectors with the goal of reducing individual and national footprints as much as possible.^26^

**Target levels of antimicrobial use**

Several antimicrobial use targets or benchmarks have been proposed in Defined Daily Doses, miligrams of antimicrobials per kilogram of animal (mg/kg), milligrams of antimicrobials per Population Correction Unity (mg/PCU).^27^

**Drug Resistance Index** (Laxminarayan et al. 2011)

The Drug Resistance Index was developed to communicate changes in the proportion of disease-causing pathogens that are resistant to commonly-used antibiotics over time. The Index combines measurements of antibiotic consumption and resistance across multiple pathogen–organism combinations to create a single metric that represents an aggregate level of drug resistance, allowing for a global assessment of the relative efficacy of countries’ antibiotic therapy.^28^

The proposed **Drug Resistance Index** was developed to communicate changes in the proportion of disease-causing pathogens that are resistant to commonly-used antibiotics over time. The Index combines measurements of antibiotic consumption and resistance across multiple pathogen-organism combinations to create a single metric^30^ that attempts to synthesize a composite human health indicator of AMR in a given country. However, it is currently only adapted for the human health sector and there is no established threshold that would allow for communicating success stories. Compared by the authors to a stock market index, the index itself is still overly technical to be adopted as a public communication tool particularly since a low Drug Resistance Index does not necessarily imply low levels of antimicrobial resistance.^31^

Another proposed idea, the **antibiotic footprint** ^32^ is more conceptually accessible and communicated to the general public as it builds on the well-known concept of the “carbon footprint.” Originally proposed as a global tool for communicating the scale of antibiotic use in humans, animals, and industry, the goal is to reduce antimicrobial use to a minimum. By design, it is a multi-sectoral indicator that synthesizes data from across different aspects of antimicrobial use, which aligns with our second criterion. However, creating a footprint for each country or as an individual footprint would violate our first criterion by incentivizing the ranking countries and assigning blame. The concept has also faced criticism, as antimicrobial use has numerous human and animal health and welfare benefits, and, unlike a carbon footprint, reducing the antibiotic footprint to zero is not a desirable goal.^33^ In addition, much like its “carbon footprint” inspiration, the concept of a personal antibiotic footprint creates a narrative of individual responsibility which reinforces misconceptions about the nature of antimicrobial resistance and reduces the focus on systemic drivers of antimicrobial use and resistance. Finally, in areas where access to antimicrobials is still a larger problem than inappropriate antimicrobial use, increases in the antimicrobial footprint might be seen as a negative, even where these increases represent necessary improvements in access.

## Embedding a Global AMR Target within the Pandemic Instrument Would Act as a Helpful Catalyst for Action

Selection of a unifying global target for AMR should be guided by the technical and political criteria considered above. Fortunately, the proposed pandemic instrument^34^ — with its focus on prevention, preparedness and response — represents an ideal opportunity to develop and adopt a unifying global target that catalyzes global action on AMR. International law represents the strongest commitment mechanism for achieving collective action on global health threats, and the proposed pandemic instrument can provide the necessary framework to promote accountability for global commitments on AMR and other related pandemic threats.^35^ In addition to a unifying global target, the pandemic instrument could enshrine other mechanisms borrowed from the Paris Climate Agreement, including a regular scientific stocktake, and a requirement for countries to make nationally determined and legally binding commitments to action in support of the global goal.^36^ The pandemic instrument could also task an independent group — such as the proposed Independent Panel on Evidence for Action on AMR^37^ — with developing these regular status reports on progress (or lack thereof) towards the global goal.

The creation of a unifying global target also creates opportunities to catalyze action to reduce inequity within global AMR action. The translation of global aspirations into national policies requires more than just setting a goal; it also requires countries to have sufficient resources and capacity to implement the actions needed to achieve the goal. This can be particularly challenging in low- and middle-income countries, where a one-size-fits-all solution may be more burdensome than in high-income countries. Failure to recognize this reality may disincentivize countries from participating in a global effort for which the costs may appear to outweigh the benefits. While some high-income countries have included funding for AMR within their international development programs, their aims are not globally coordinated and insufficient to resource a truly global effort. Articulating a unifying global target creates an opportunity to align funding and capacity building supports in service of the broader global goal.

## Conclusion

The proposed pandemic preparedness instrument represents an ideal opportunity to harness the power of international law to establish a unifying global target that catalyzes global action on AMR. While the majority of proposed AMR targets and indicators to date have been too technical to succeed as a unifying global target, the creation of a target that could act as a barometer of global progress across sectors, synthesize technical indicators into a single clearly communicated concept, and offer identifiable moments of success would represent a monumental step forward towards catalyzing collective action on AMR.

## Note

This work was supported by the Canadian Institutes of Health Research [#149542], the Social Sciences & Humanities Research Council [#895-2022-1015], and the Wellcome Trust [222422/Z/21/Z]. The funding bodies were not involved in the study design, data collection, analysis, interpretation or writing.

## References

[1] WHO, Global Action Plan on Antimicrobial Resistance, 2015, *available at* < https://apps.who.int/iris/bitstream/handle/10665/193736/9789241509763_eng.pdf?sequence=1> (last visited October 26, 2022).

[2] S. Rogers Van Katwyk, M. Balasegaram, P. Boriello P, et al., “A Roadmap for Sustainably Governing the Global Antimicrobial Commons,” The Lancet 394 (2019): 1788–9. 10.1016/S0140-6736(19)32767-9 31741444

[3] S. Tejpar , S. Rogers Van Katwyk , L. Wilson , and S.J. Hoffman , “Taking Stock of Global Commitments on Antimicrobial Resistance,” BMJ Global Health 7 (2022): e008159. 10.1136/bmjgh-2021-008159 PMC9121412 35589150

[4] C.J. Murray , K.S. Ikuta , F. Sharara , et al., “Global Burden of Bacterial Antimicrobial Resistance in 2019: A Systematic Analysis,” The Lancet (2022): S0140673621027240. 10.1016/S0140-6736(21)02724-0 PMC8841637 35065702

[5] C.I.R. Chandler , “Current Accounts of Antimicrobial Resistance: Stabilisation, Individualisation and Antibiotics as Infrastructure,” Palgrave Communications 5 (2019): 1–13. 10.1057/s41599-019-0263-4 PMC6542671 31157116

[6] UNAIDS, *90-90-90: An Ambitious Treatment Target to Help End the AIDS Epidemic*, 2014.

[7] UNFCCC, *Paris Agreement*, 2015, *available at* <http://unfccc.int/resource/docs/2015/cop21/eng/l09r01.pdf> (last visited October 26, 2022).

[8] T. Epton and C.J. Armitage , “Goal Setting Interventions,” in M.S. Hagger , L.D. Cameron , K. Hamilton , N. Hankonen , and T. Lintunen , eds., The Handbook of Behavior Change, 1st ed. (Cambridge: Cambridge University Press, 2020): at 554–71.

[9] *Id.*

[10] F. Biermann , N. Kanie , and R.E. Kim , “Global Governance by Goal-Setting: The Novel Approach of the UN Sustainable Development Goals,” Current Opinion in Environmental Sustainability 26–27 (2017): 26–31; A. Broome and J. Quirk , “Governing the World at a Distance: The Practice of Global Benchmarking,” *Review of International Studies* 41 (2015): 819–41.

[11] J.P.G. Jones , B. Collen , G. Atkinson , et al., “The Why, What, and How of Global Biodiversity Indicators beyond the 2010 Target: Biodiversity Indicators Beyond 2010,” Conservation Biology 25 (2011): 450–7. 21083762 10.1111/j.1523-1739.2010.01605.x

[12] S. Fukuda-Parr , “Global Goals as a Policy Tool: Intended and Unintended Consequences,” Journal of Human Development and Capabilities 15 (2014): 118–31.

[13] G.M. Mace , W. Cramer , S. Díaz , et al., “Biodiversity Targets after 2010,” Current Opinion in Environmental Sustainability 2 (2010): 3–8.

[14] S. Fukuda‐Parr and D. McNeill , “Knowledge and Politics in Setting and Measuring the SDGs: Introduction to Special Issue,” Global Policy 10 (2019): 5–15.

[15] *Id.*

[16] J. Sachs , “From Millennium Development Goals to Sustainable Development Goals,” The Lancet 379 (2012): 2206–11. 10.1016/S0140-6736(12)60685-0 22682467

[17] W. Easterly , “How the Millennium Development Goals Are Unfair to Africa,” World Development 37 (2009): 26–35.

[18] See Sachs, *supra* note 15.

[19] S. Rogers Van Katwyk , A. Giubilini , C. Kirchhelle , et al., “Exploring Models for an International Legal Agreement on the Global Antimicrobial Commons: Lessons from Climate Agreements,” *Health Care Analysis* (2020), *available at* <https://link.springer.com/article/10.1007/s10728-019-00389-3> (last visited October 26, 2022). 10.1007/s10728-019-00389-3 PMC10042908 31965398

[20] A. Weinberg , “The ‘Flatten the Curve’ Chart Was Ugly and Not Exactly Rigorous. Here’s Why It Worked So Well,” *Mother Jones* (2020), *available at* <https://www.motherjones.com/media/2020/06/flatten-the-curve-chart-infographics/> (last visited October 26, 2022).

[21] See UNFCC, *supra* note 7.

[22] See Murray et al., *supra* note 4; J. O’Neill, *Tackling Drug-Resistant Infections Globally: Final Report and Recommendations—The Review on Antimicrobial Resistance*, 2016, *available at* <https://amr-review.org/sites/default/files/160525_Final%20paper_with%20cover.pdf> (last visited October 26, 2022).

[23] M. Mendelson , M. Balasegaram , T. Jinks , C. Pulcini , and M. Sharland , “Antibiotic Resistance Has a Language Problem,” Nature 545 (2017): 23-5; *Reframing Resistance: How to Communicate about Antimicrobial Resistance Effectively*, 2019, *available at* <https://wellcome.org/sites/default/files/reframing-resistance-report.pdf> (last visited October 26, 2022). 10.1038/545023a 28470219

[24] J. Santos , “Reflections on the Impact of ‘Flatten the Curve’ on Interdependent Workforce Sectors,” Environment Systems and Decisions 40 (2020): 185–8. 32421098 10.1007/s10669-020-09774-z PMC7224345

[25] T.W. O’Rourke , “Applying the Concepts of ‘Community Spread’ and ‘Flatten the Curve’ to Chronic Conditions and Their Prevention,” American Journal of Health Education 51 (2020): 199–202.

[26] D. Limmathurotsakul , J.A.T. Sandoe , D.C. Barrett , et al., “’Antibiotic Footprint’ as a Communication Tool to Aid Reduction of Antibiotic Consumption,” Journal of Antimicrobial Chemotherapy 74 (2019): 2122–7. 31074489 10.1093/jac/dkz185 PMC6640305

[27] F.M. MacKenzie and I.M. Gould , “Quantitative Measurement of Antibiotic Use,” in I.M. Gould , J.W.M. van der Meer , eds., Antibiotic Policies (Boston: Springer, 2005): at 105–18; D. Góchez, M. Raicek, J. Pinto Ferreira, M. Jeannin, G. Moulin, E. Erlacher-Vindel, “OIE Annual Report on Antimicrobial Agents Intended for Use in Animals: Methods Used,” *Frontiers in Veterinary Science* 6 (2019): 317; T.P. Van Boeckel, S. Gandra, A. Ashok, et al., “Global Antibiotic Consumption 2000 to 2010: An Analysis of National Pharmaceutical Sales Data,” *The Lancet Infectious Diseases* 14, no. 8 (2014): 742-50.

[28] R. Laxminarayan and K.P. Klugman , “Communicating Trends in Resistance Using a Drug Resistance Index,” BMJ Open 1 (2011): e000135–e000135; E.Y. Klein, K.K. Tseng, S. Pant, and R. Laxminarayan, “Tracking Global Trends in the Effectiveness of Antibiotic Therapy Using the Drug Resistance Index,” *BMJ Glob Health* 4 (2019): e001315. 10.1136/bmjgh-2018-001315 PMC6509601 31139449

[29] See *supra* note 27.

[30] See *supra* note 28.

[31] C.M.J.E. Vandenbroucke-Grauls , G. Kahlmeter , J. Kluytmans , et al., “The Proposed Drug Resistance Index (DRI) Is Not a Good Measure of Antibiotic Effectiveness in Relation to Drug Resistance,” BMJ Global Health 4 (2019): e001838. 10.1136/bmjgh-2019-001838 PMC6730578 31543998

[32] See *supra* Limmathurotsakul et al., note 26.

[33] M. Lipsitch and J. Shaman , “Comment on: ‘Antibiotic Footprint’ as a Communication Tool to Aid Reduction of Antibiotic Consumption,” Journal of Antimicrobial Chemotherapy 74 (2019): 3404–6. 31314102 10.1093/jac/dkz320

[34] *Working Draft, Presented on the Basis of Progress Achieved, for the Consideration of the Intergovernmental Negotiating Body at Its Second Meeting*, 2022, published online July 13, 2022.

[35] See Rogers Van Katwyk et al., *supra* note 19; S.J. Hoffman , J.-A. Røttingen , and J. Frenk , “Assessing Proposals for New Global Health Treaties: An Analytic Framework,” American Journal of Public Health 105 (2015): 1523–30; S.J. Hoffman, R. Bakshi, and S.R.V. Katwyk, “How Law Can Help Solve the Collective Action Problem of Antimicrobial Resistance,” *Bioethics* 33, no. 7 (2019): 798-804; A. Behdinan and S.J. Hoffman, “Towards an International Treaty on Antimicrobial Resistance,” *Ottawa Law Review* 47 (2016): 507-32.26066926

[36] *Id.* (Rogers Van Katwyk et al.).

[37] Independent Panel on Evidence for Action Against Antimicrobial Resistance Final Draft Terms of Reference for Public Discussion, available at <https://global1hn.ca/wp-content/uploads/2020/06/Evidence-panel-Action-against-AMR.pdf> (last visited October 27, 2022).

